# Biologically Active Compounds of Plants: Structure-Related Antioxidant, Microbiological and Cytotoxic Activity of Selected Carboxylic Acids

**DOI:** 10.3390/ma13194454

**Published:** 2020-10-08

**Authors:** Beata Godlewska-Żyłkiewicz, Renata Świsłocka, Monika Kalinowska, Aleksandra Golonko, Grzegorz Świderski, Żaneta Arciszewska, Edyta Nalewajko-Sieliwoniuk, Monika Naumowicz, Włodzimierz Lewandowski

**Affiliations:** 1 Department of Analytical Chemistry, Faculty of Chemistry, University of Bialystok, K. Ciołkowskiego 1K, 15–245 Białystok, Poland; z.arciszewska@uwb.edu.pl (Ż.A.); e.nalewajko@uwb.edu.pl (E.N.-S.); 2 Department of Chemistry, Biology and Biotechnology, Bialystok University of Technology, Wiejska 45E, 15–351 Białystok, Poland; r.swislocka@pb.edu.pl (R.Ś.); m.kalinowska@pb.edu.pl (M.K.); g.swiderski@pb.edu.pl (G.Ś.); 3 Institute of Agricultural and Food Biotechnology, Rakowiecka 36, 02–532 Warsaw, Poland; olau.95@gmail.com; 4 Department of Physical Chemistry, Faculty of Chemistry, University of Bialystok, K. Ciołkowskiego 1K, 15–245 Białystok, Poland; monikan@uwb.edu.pl

**Keywords:** phenolic acids, natural carboxylic acids, structure-activity relationship, hydroxyl groups, antibacterial, antioxidant, cytotoxic activity

## Abstract

Natural carboxylic acids are plant-derived compounds that are known to possess biological activity. The aim of this review was to compare the effect of structural differences of the selected carboxylic acids (benzoic acid (BA), cinnamic acid (CinA), p-coumaric acid (*p*-CA), caffeic acid (CFA), rosmarinic acid (RA), and chicoric acid (ChA)) on the antioxidant, antimicrobial, and cytotoxic activity. The studied compounds were arranged in a logic sequence of increasing number of hydroxyl groups and conjugated bonds in order to investigate the correlations between the structure and bioactivity. A review of the literature revealed that RA exhibited the highest antioxidant activity and this property decreased in the following order: RA > CFA ~ ChA > *p*-CA > CinA > BA. In the case of antimicrobial properties, structure-activity relationships were not easy to observe as they depended on the microbial strain and the experimental conditions. The highest antimicrobial activity was found for CFA and CinA, while the lowest for RA. Taking into account anti-cancer properties of studied NCA, it seems that the presence of hydroxyl groups had an influence on intermolecular interactions and the cytotoxic potential of the molecules, whereas the carboxyl group participated in the chelation of endogenous transition metal ions.

## 1. Introduction

Plants are a natural source of functionally active compounds. Various aromatic plants for centuries have been used as preservatives in foods. Phytochemicals are plant secondary metabolites with the primary function of chemical defence against insects and microorganisms. Many bioactive substances such as phenols, flavonoids, quinones, coumarins, phenolic acids, tannins, terpenes and alkaloids belong to this category. These compounds frequently have free radical scavenging potential due to their stable structure after trapping the free radical. Several reports on the antimicrobial activity of phytochemicals isolated from herbs, such as thymol, carvacrol, linalool, *trans*-caryophyllene, *p*-cymen, and *γ*-terpinene, and phenolic acids, such as gallic, rosmarinic, protocatechuic or caffeic, have been published [1,2,3]. Moreover, numerous phenolic compounds possess also antiviral, anti-obesity, anti-diabetic, and anti-inflammatory properties as well as anti-cancer effect through their antioxidant property [4,5]. It is generally accepted that compounds having the hydroxyl group attached to a phenyl ring have the greatest antimicrobial activity among the secondary metabolites found in plants. It has been also suggested that the aromaticity is also responsible for this effect. Therefore, for purpose of this review several natural carboxylic acids (NCA) arranged in a logic sequence of increasing number of hydroxyl groups and conjugated bonds have been selected, namely: benzoic acid (BA), cinnamic acid (CinA), *p*-coumaric acid (*p*-CA), caffeic acid (CFA), rosmarinic acid (RA) (an ester of caffeic acid and 3, 4-dihydroxyphenyllactic acid), and chicoric acid (ChA) (an ester of two caffeic acids and tartaric acid).

The health-promoting properties of phenolic acids contained in plants were appreciated already in antiquity and used in folk medicine [6]. Despite the passage of years, this group of compounds is still the subject of much research on their unique antibacterial, cardioprotective, anti-inflammatory, and anti-cancer properties. Recent studies also focus on explaining the relationship between dietary polyphenols and phenolic acids and memory impairment [7], antidepressant effects [8], and retinal degeneration [9]. Last year’s reports described several interesting new properties of the analysed acids, including inhibiting the activity of *p*-coumaric acid on interbacterial quorum-sensing communication, which may be an innovative solution in the preservation of perishable food such as meat [10]. The team of Wang et al. [11] created polymer nanoparticles containing caffeic acid derivatives with bacteriostatic properties against bacterial plant pathogens that could potentially replace conventional pesticides. Furthermore, Chung et al. [12] observed that PEGylated rosmarinic acid nanoparticles alleviated acute inflammatory bowel disease in a mouse model in vivo. Such interesting results motivate us to take a closer look at the structure of these compounds. The dependencies are not that easy to explain. An additional difficulty results from individual differences, such as the composition of the intestinal microflora, which determines the concentration of polyphenols that enter the bloodstream. According to the research conducted by Monagas et al. [13] intestinal microbiota is responsible for the breakdown of polyphenols into low molecular weight polyphenol conjugates and phenolic acids that can be absorbed. Therefore, the observed systemic effects may result from the action of phenolic acids as metabolites of polyphenols, and not strictly parent phenolic compounds ingested with food.

Therefore, the aim of this review is to summarize and evaluate the physicochemical properties of selected compounds naturally occurring in plants, their potential of antioxidant behaviour and microbiological activity. A series of carboxylic acids that possess conjugate bond with increasing number of hydroxyl groups in molecule: cinnamic acid, p-coumaric acid and caffeic acid, as well as the series of carboxylic acids with increasing number of conjugate bonds: Benzoic acid, cinnamic acid, rosmarinic acid, and chicoric acid were selected in order to find and formulate a general relationship between the molecular structure and the biological activity, as it is believed that these properties possibly could be correlated with their antimicrobial and anticancer activity. The undertaken analysis is part of a larger project in which we are looking for structural differences between individual phenolic compounds that are responsible for the visible biological effect. Our research is not only aimed at finding the correlation between the structure and biological activity, but also some application possibilities, such as the creation of safe antimicrobial agents, also in food preservation. In order to create targeted modifications of the structure enhancing its activity, it is; therefore, necessary to thoroughly understand the mechanisms of action of a given molecule under systemic conditions. It is known that even small changes in the structure can radically change the electron charge in the molecule, and thus the ability to react with biological macromolecules and free radicals.

## 2. Chemical Structure, Occurrence, and Physicochemical Parameters of NCA

**Figure 1:**
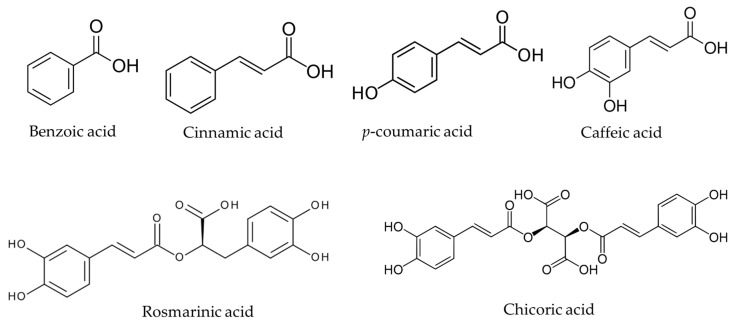
Chemical structures of reviewed natural carboxylic acids (NCA): benzoic acid (BA), cinnamic acid (CinA), *p*-coumaric acid (*p*-CA), caffeic acid (CFA), rosmarinic acid (RA), and chicoric acid (ChA).

The chemical structures of the reviewed natural carboxylic acids are presented in Figure 1. Benzoic acid (BA) is the simplest aromatic monocarboxylic acid comprising a benzene ring core with a carboxylic acid substituent. Cinnamic acid (CinA) ((E)--3-phenylprop-2-enoic acid) is an unsaturated monocarboxylic acid comprising an acrylic acid bearing a phenyl substituent at the 3-position. It occurs as both *cis* and *trans* isomer, although the *trans* form is more common. It is a precursor for the synthesis of a huge number of other more complex phenolic compounds. *p*-coumaric acid (*p*-CA) (4-hydroxycinnamic acid) is one of the three hydroxyl derivatives of cinnamic acid that differ by the position of the hydroxy substitution of the phenyl group. Caffeic acid (CFA) ((E)-3-(3,4-dihydroxyphenyl)prop-2-enoic acid, 3,4-dihydroxycinnamic acid) is also a hydroxyl derivative of CinA in which the phenyl ring is substituted by hydroxyl groups at positions 3 and 4. It exists in *cis* and *trans* forms, although the latter is more common. CFA is the building block of a variety of the plant metabolites from the simple monomers to multiple condensation products giving a variety of caffeic acid derivatives. Rosmarinic acid (RA) is an ester of caffeic acid and 3,4-dihydroxyphenyllactic acid and its chemical structure contains five hydroxyl groups. Chicoric acid (ChA) (also known as cichoric acid) ((2*R*,3*R*)-2,3-bis[[(E)-3-(3,4-dihydroxyphenyl) prop-2-enoyl]oxy]butanedioic acid) is a tartaric acid ester of two caffeic acids. It possesses six hydroxyl groups in the structure; its most abundant natural form is L-chicoric acid.

Natural carboxylic acids can be found in all plant tissues, including edible parts such as fruits, seeds, leaves, stems, and roots. BA and CinA are naturally present in fruits, vegetables, nuts, herbs, spices, as well as fungal and animal tissues. BA can be also produced by microorganisms during food processing. *Cinnamomum cassia* (L.) J. Presl–called chinese cinnamon-is the richest natural sources of BA (0.336 mg/g) and CinA (0.01–1.91 mg/g) [14]. The content of BA in sage (*Salvia officinalis*), thyme (*Thymus vulgaris*), and nutmeg is in the range of 0.015–0.05 mg/g. Other spices, such as turmeric, coriander, laurel, paprika, and white and black pepper contain lower amounts of this compound (0.001–0.005 mg/g) [15]. CinA can be also found in citrus fruits, grapes, tea, cocoa, spinach, celery, and brassicas vegetables [16]. Phenolic acids, such as *p*-coumaric, caffeic, and rosmarinic acids occur in small amounts in almost all green plants: fruits, vegetables, herbs, grains, and mushrooms. They are frequently present in herbs and spices, such as rosemary, thyme, oregano, sage, cinnamon, cumin, and bay. *p*-CA serves as a precursor of other phenolic compounds and exists either in free or conjugated forms in plants. It forms conjugates with mono-, oligo-, and polysaccharides, alkyl alcohols, organic acids, amine, and lignin. The content of free *p*-CA is very high in some mushroom species (traditional Chinese medicines) and vary from several milligrams per gram to nearly a thousand times higher than that in fruits and herbs [17,18]. The level of *p*-CA determined in cinnamon, thyme, oregano, and rosemary was in the range 0.0022–0.0096 mg/g of dry weight (DW). CFA is widely distributed in plant tissues. Coffee is the primary source of CFA in the human diet. Other edible plants that have been found to contain CFA include sweet potatoes and artichoke. This polyphenol is present in many other food sources, including blueberries, apples and cider, olive oil, and many culinary herbs: caraway, thyme, oregano, and rosemary. The high content of CFA and *p*-CA was found in sage (1.215 mg/g DW) and oregano (2.148 mg/g DW) [19]. Lower amounts are present in bay, marjoram, and cinnamon [20]. RA is naturally occurring in several plants of the Lamiaceae family, including rosemary, from which it was originally isolated, sage and Spanish sage, basil, oregano, marjoram, and lemon balm. In lower amounts it was found in bay, cinnamon, and cumin [21]. ChA is most often reported in the family *Asteraceae* (Aster family), or the family *Dryopteridaceae* (Wood fern family). *Cichorium intybus* and *Echinacea purpurea* (L.) *Moench.* are well-known for their ChA production. But it was identified also in other plants (25 families, 63 genera and species) [22]. The concentration and the composition of phytochemicals vary significantly among different plant species, due to genotypic and environmental variability within the species (Table 1). They can also vary greatly between cultivars and different parts of the same plant. For example ChA content in 15 basil cultivars ranged from 0.03 to 2.78 mg/g DW [23]. Lee and Scagel reported ChA allocation of flowering *E. purpurea*, as leaves > roots > flowers > stems, and its content was in the range 0.93–3.91 mg/g fresh weight of fraction [24]. Apart from the biological variety, different content of bioactive compounds determined in plants could be also a result of using various solvent extraction protocols for their recovery from the plant raw materials. The efficiency of extraction of plant materials is mostly based on the correct choice of solvents and the use of heat and/or agitation to increase the solubility of materials and the rate of the mass transfer. Among a huge variety of solvents, water, methanol, and ethanol or their mixtures are most often used for extraction of phytochemicals (Table 1). Therefore, the values presented in Table 1 should be assumed as informative only. Nevertheless, it is widely accepted that aromatic plants and spices are a rich source of bioactive compounds for human diet.

In aqueous solutions, carboxylic acids exist in molecular and deprotonated forms. Table 2 depicts thermodynamic dissociation constants of the carboxyl and hydroxyl groups of the reviewed NCA.

From experimental data and calculated values one can conclude that the molecular and monodeprotonated forms of these acids prevail in the region of physiological pH values. The corresponding dissociation constants values are similar which indicates that this parameter is apparently not a major determining factor for the activity of the carboxylic acids and that other physicochemical properties could control their antimicrobial or antioxidant activity. Nevertheless, these data could be a useful tool for bioavailability and pharmacokinetics studies because some of these compounds are intrinsic components of diet [35].

Logarithmic coefficient of substance distribution (log *p*) in the water–n-octanol system (Table 2) is used as important tool in studies of the environmental fate of chemicals as a measure of their hydrophilicity/lipophilicity. Initially, it was considered in drug and pesticide discovery and design, but now it is an important characteristic of any chemical because it determines to a large extent a chemical’s fate both inside a living organism and in the environment. For example, biological properties such as bioaccumulation and toxicity are largely determined by log *p* [57]. The log *p* values collected for all analysed carboxylic acids suggest that these compounds are moderately hydrophobic (1 < log *p* < 3) [47].

Log *p*, together with molecular weight (*M*_W_), H-bond acceptor (HBA), and H-bond donor (HBD), are useful in determination of basic pharmacokinetic properties of bioactive compounds (Table 2). Restrictions for using the *M*_W_, log *p*, HBA, and HBD were stipulated by Lipinski’s rule of five (also known as the rule of five) [58]. The rule of five is a general rule to evaluate drug likeness or determine if a chemical compound with a certain pharmacological or biological activity has properties that would make it a likely orally active drug in humans [54]. The rule predicts that an orally active drug has no more than one violation of the following criteria: a molecular mass less than 500 daltons, log *p* that does not exceed 5, no more than five hydrogen bond donors (the total number of nitrogen–hydrogen and oxygen–hydrogen bonds) and no more than 10 hydrogen bond acceptors (all nitrogen or oxygen atoms) [58]. From the data displayed in Table 2, all compounds meet the requirement of Lipinski’s rule.

## 3. Antioxidant Activity of NCA Determined by Various Chemical Methods

Reactive oxygen species (ROS) and reactive nitrogen species (RNS) are chemically active molecules produced during metabolic processes in cells. As a result of disturbance of redox homeostasis, the state of equilibrium in the body between the amount of ROS or RNS and the concentration of endogenous substances with antioxidant properties, lipids, proteins and nucleic acids may be damaged. A consequence of “oxidative stress” is overproduction of free radicals which are involved in the development of various civilization diseases: diabetes, atherosclerosis, hypertension, neurodegenerative diseases, and even some types of cancer [59]. Dietary antioxidants are substances that support the internal antioxidant defence system and help to prevent oxidative stress-related disorders. They can act as reducing agents, hydrogen donors, singlet oxygen quenchers or metal chelating agents [60,61]. Two major factors that determine the mechanism and the efficacy of antioxidants are bond dissociation energy (BDE) and ionization potential (IP) of the molecule.

To assess the antioxidant activity a variety of different methods are used, such as 2,2-diphenyl-1-picrylhydrazyl (DPPH) radical scavenging assay, 2,2′-azinobis(3-ethylbenzothiazoline-6-sulfonic acid) radical cation (ABTS^•+^) scavenging assay, ferric reducing of antioxidant power (FRAP) assay, cupric ion reducing antioxidant capacity (CUPRAC) method, the total radical-trapping antioxidant parameter (TRAP) assay, oxygen radical absorbance capacity (ORAC) assay, Trolox (6-hydroxy-2,5,7,8-tetramethylchroman-2-carboxylic acid) equivalent antioxidant capacity (TEAC) method, inhibition of lipid peroxidation, ferric thiocyanate method, hypochlorous acid, hydrogen peroxide, superoxide anion and hydroxyl radical scavenging assays, or superoxide dismutase activity (SOD) assay. The antioxidant activity is usually expressed as the relative radical scavenging activity (RSA) in percent and calculated by using the following formula: (A_B_ − A_A_)/A_B_ × 100 (where A_B_ is the absorbance of the radical solution without the sample; A_A_ is the absorbance of the radical solution in the presence of an antioxidant). Radical scavenging activity is also expressed as IC_50_ value, which means the concentration of the tested antioxidant that shows a 50% inhibitory effect. In ABTS, DPPH, ORAC, CUPRAC or FRAP assays results are also reported as Trolox equivalent antioxidant capacity (TEAC). TEAC value is defined as the millimolar concentration of a Trolox solution whose antioxidant capacity is equivalent to a 1.0 mmol/L solution of the tested antioxidant [62].

Methods used to assess the antioxidant activity can be generally divided into two groups based on the mechanism of deactivation of free radicals: Hydrogen atom transfer (HAT) and single electron transfer (SET). HAT-based methods (e.g., the oxygen radical absorbance capacity (ORAC) [63] and the total radical trapping antioxidant potential (TRAP) [64]), measure the classical ability of an antioxidant to quench free radicals by hydrogen donation. HAT reactions are solvent and pH independent and are usually quite rapid, typically completed in s to min. SET-based methods (e.g., the ferric reducing antioxidant power (FRAP) [65] or the CUPRAC (cupric ion reducing antioxidant capacity) [66]), detect the ability of a potential antioxidant to transfer one electron to reduce any compound, including metals, carbonyls, and radicals. SET reactions are usually slow and can require long times to reach completion, so antioxidant capacity calculations are based on percent decrease in product rather than kinetics. Some analytical methods cannot be unequivocally qualified into none of the above groups, namely TEAC [67] or DPPH. The mechanisms of the above mentioned methods, as well as their advantages and limitations, are discussed in a comprehensive review of Prior et al. [68].

### 3.1. Scavenging of the DPPH Radical

The DPPH radical is a stable, synthetic, free radical which accepts hydrogen radical or an electron and become a stable diamagnetic molecule. In order to evaluate antioxidant activity, methanolic solution of DPPH^•^ is mixed with solution containing antioxidant. The samples are incubated for 2–60 min in the dark at room temperature; the decrease in the absorbance of the resulting solution is measured at 515–520 nm [69]. Table 3 presents the scavenging activity of natural carboxylic acids on the DPPH radical. It could be seen, that BA did not exhibited radical scavenging capacity [70] or it was very low (about 4%) [71]. CinA and *p*-CA showed weak to moderate radical scavenging activity from 2.1% to 62.5% [72] and from 5.3% to 55.6% [73], with the minimal IC_50_ values equal 160 [74] and 3.96 µmol/L [75], respectively. However, Villaño et al. [62] indicated that the scavenging capacity of *p*-CA was not significant. The radical scavenging activities were considerably higher when a catechol group was present in the chemical structure of compound (CFA, RA, ChA). CFA exhibited from 28.5% to 93.6% free radical removal capability [76], the minimal IC_50_ value was 0.17 µmol/L [77]. RA is a naturally occurring caffeic acid ester with health promoting effects [78]. It contains four hydroxyl groups which are responsible for its high antioxidative capacity (Table 3). The lowest IC_50_ value reported for RA was 0.5 µmol/L [79]. A review of the literature shows that there are only few papers on the antioxidant activity of ChA (Table 3), which exhibited about 56% DPPH^•^ scavenging activity [80] and the minimal IC_50_ value equal 8.6 µmol/L [81]. It is difficult to compare the data obtained by different researchers due to using by them different measurement procedures.

However, there are papers in which antioxidative capacity of several carboxylic acids was tested and compared under the same measurement conditions. The results obtained by Takahashi and Miyazawa [82] showed that the DPPH radical scavenging activity order was: CFA > *p*-CA = CinA. Also data presented by other authors revealed that CFA was stronger antioxidant than *p*-CA [62,75,77,83,84]. While Szwajger et al. [70] found that the DPPH free radical removal capability of the three investigated carboxylic acids was in the order: CinA > *p*-CA > BA with inhibition efficiency values of 35%, 27%, and 0%, respectively. The scavenging effect increased with increasing concentration of antioxidants [83]. Velkov et al. [85] investigated scavenging capacity of twenty compounds including CinA and its derivatives versus DPPH radical and found that their activity was in the order: RA > CFA > *p*-CA > CinA with RSA values of 88.4%, 76.6%, 3.6%, and 0.5%, respectively. Moreover, RA and CFA exhibited higher RSA values than well-known antioxidants such as: tert-butylhydroquinone (TBHQ) (58.7%), α-tocopherol (54.0%), Trolox (53.4%), butylated hydroxyanisole (BHA) (22.3%) or butylated hydroxytoluene (BHT) (8.0%).

### 3.2. Scavenging of the ABTS Radical

In this assay, ABTS is oxidized to its blue-green radical cation (ABTS^•+^) usually by 2,2′-azobis(2-amidino-propane)dihydrochloride (AAPH) or potassium persulfate (K_2_S_2_O_8_) [86]. Antioxidant activity is measured as the ability of antioxidant to inhibit the absorbance of the ABTS radical chromogen, which has a characteristic long-wavelength absorption spectrum at 734 nm. ABTS assay is applicable for both hydrophilic and lipophilic antioxidants. The reaction with ABTS^•+^ is quite fast, the reaction times ranging from 1 to 30 min. The scavenging activities of the studied natural carboxylic acids on the ABTS radical cation are presented in Table 3. CinA exerted no antioxidant activity due to its negative hydrogen-donating ability [82,87,88,89]. Additionally, BA did not show radical scavenging activity against ABTS^•+^ radical cation [70] or it was extremely low (<1%) [90]. *p*-CA revealed moderate radical scavenging activity equal 51.7% [84], and its minimal IC_50_ value reported by Singh et al. [91] was 50.0 µmol/L. However, Villaño et al. [62] indicated that *p*-CA did not show the scavenging activity against ABTS^•+^. Similarly to the DPPH method, phenolic acids with a catechol group present in their chemical structure demonstrated high antioxidant activity. CFA exhibited from 32.1% to 92.9% free radical removal capability [76,84,92] with the minimal IC_50_ value equal 17.5 µmol/L [93]. The IC_50_ value reported for RA was 2.91 µmol/L [94]. ChA exhibited 49.1% free radical cation removal capability. A review of the literature showed that there are only a few papers in which antioxidant capacity of two out of six carboxylic acids described in this work were tested under the same experimental conditions. The results obtained by Masek et al. [84] and Singh et al. [91] showed that *p*-CA exhibited higher ABTS radical scavenging activity comparing to CFA. However, these results are in contrary to the results obtained by Villaño et al. [62], who found that CFA demonstrated higher antioxidant activity than *p*-CA.

### 3.3. Ferric Reducing Antioxidant Power (FRAP) Assay

This method is based on the ability of antioxidants to reduce Fe^3+^ to Fe^2+^ ions in the presence of 2,4,6-tripyridyl-s-triazine (TPTZ). Within the reaction, the colourless Fe(III)-TPTZ complex is converted into the blue-coloured Fe(II)-TPTZ complex and the change in the absorbance at 593 nm, that directly reflects the TAC of sample, is measured spectrophotometrically. Ferric reducing power could be expressed as IC_50_ value, in % or reported as Fe^2+^ equivalents. The FRAP reaction is carried out at acidic solution (pH 3.6) to maintain iron solubility. In such environment the ionization potential that drives hydrogen atom transfer decreases and increases the redox potential, which is the dominant reaction mechanism. Because the reaction detects compounds with redox potentials of <700 mV, which is comparable with that of ABTS^•+^ (680 mV), similar compounds react in both the ABTS and FRAP assays [68]. FRAP cannot detect compounds that act by radical quenching (hydrogen transfer), particularly thiols (as glutathione).

However, FRAP is simple, rapid (generally 4–6 min), inexpensive, and can be performed using semiautomatic or automated protocols. Mathew et al. [71] used this approach to test reducing potential of sixteen compounds including BA and CFA. They found very low reducing power of BA, whereas CFA showed a strong reducing potential. Jitareanu et al. [95] positioned the reducing potential of the NCA compounds in the following order: CFA > *p*-CA > CinA. Similar results were obtained by Masek et al. [84] after comparing the reduction activity of *p*-CA (4.6%) and CFA (30.8%) at their equivalent concentration of 30 µg/mL. They also reported that increasing concentration of the tested compounds resulted in their higher reduction activity. The effect of the dose on the reducing power of CFA and ChA was also observed by Liu et al. [80]. Their results showed that the FRAP value of CFA was significantly lower than that of ChA at low concentrations (10–100 µmol/L); however, its reducing power was higher than ChA at concentrations in the range of 250–500 µmol/L. The above results demonstrate that ferric reducing power of antioxidants depends on the concentration of compound [80,84] the degree of hydroxylation, and the extent of conjugation of antioxidant [68]. When comparing RA, CinA, *p*-CA, and CFA, the presence of an additional hydroxyl group increases reducing activity.

### 3.4. The CUPRAC (Cupric Reducing Antioxidant Capacity) Method

CUPRAC was originally developed for determination of the total antioxidant activity level in extracts of plants. It is a variant of the FRAP assay, that is based on the reduction of Cu^2+^ to Cu^+^ (instead of Fe) by the combined action of reducing agents in the sample. Bathocuproine (2,9-dimethyl-4,7-diphenyl-1,10-phenanthroline) or neocuproine (2,9-dimethyl-1,10-phenanthroline) are used to form chromophores with Cu^+^ that absorb at 490 or 450 nm, respectively. The use of copper has many advantages over iron in the antioxidant tests, because all classes of antioxidants, including thiols, are detected with little interference from reactive radicals, and the reaction kinetics of copper is faster than iron [68]. The method is applicable to both hydrophilic and lipophilic antioxidants. The TEAC coefficients of *p*-CA, CFA, and RA determined by the CUPRAC method are shown in Table 3.

Apak et al. [66] reported TEAC values for *p*-CA acid and CFA in ethanol solution as 0.55 and 2.8 after 30 min incubation at room temperature, and 1.00 and 2.96 after 20 min incubation at 50 °C. Yıldız et al. [115] also investigated the influence of temperature on the determined TEAC coefficients (in methanol) and found very slight effect (3–6%) for CFA and RA and strong effect on *p*-CA (3-fold increase). Examination of the CUPRAC capacity in methanol at room temperature performed by Çelik et al. [116] resulted in higher values for *p*-CA and CFA and lower for RA than reported earlier. In general, in an electron transfer-based antioxidant assay like CUPRAC, the molar absorptivity for antioxidant compounds may show certain variations depending on the composition and polarity of the solvent medium [116]. All studies [66,114,116] reported the antioxidant capacity in the CUPRAC method in following order: RA > CFA > *p*-CA.

### 3.5. The Oxygen Radical Antioxidant Capacity (ORAC)

ORAC employs a competitive reaction scheme between antioxidants and a fluorescence probe for a peroxyl radical [63]. As the fluorescent probes, fluorescein or 2′,7′-dichlorofluorescein diacetate are most often used. This assay allows to accurately measure both the inhibition time and the inhibition degree of lipophilic and hydrophilic antioxidants to ensure an accurate measurement of the antioxidant activity. The time of analysis is long as it measures the reaction until its completion and detects fast- and slow-reacting antioxidants. ORAC values were only reported for *p*-CA and CFA [117], and expressed as Trolox equivalents (Table 3). In both studies, CFA had higher ORAC values than *p*-CA and thus higher antioxidant activity. Villaño et al. [62] demonstrated that BA derivatives showed lower antioxidant activity compared to CinA derivatives. They observed the effect of the catechol group on the activity towards peroxyl radicals of both BA and CinA derivatives. Indeed, the highest ORAC values were reported for compounds containing a catechol group in their structure.

### 3.6. Lipid Peroxidation Assay (LP)

ROS may also attack polyunsaturated fatty acids of cell membranes causing the destruction of membrane lipids, that is especially dangerous for the viability of cells, or even tissues. Malondialdehyde (MDA) is one of several low-molecular weight products of lipid peroxidation. The monitoring of MDA levels in biological systems is presently regarded as an important indicator of lipid peroxidation and peroxidative tissue injury [119]. The assay is based on a condensation reaction of two molecules of 2-thiobarbituric acid (TBA) with one molecule of MDA, which is carried out at low pH and elevated temperature and results in generation of a red, fluorescent adduct. Amist and Singh [120] studied the effect of BA on MDA production and found that BA was responsible for increased MDA content. Even at the lowest tested BA concentration (0.5 mmol/L), MDA content was 9% higher than the control. Higher concentrations of BA (1 and 1.5 mmol/L) caused an increase in MDA content of about 67% compared to the control. Ekinci-Akdemir et al. [121] showed the effect of *p*-CA treatment on oxidative stress parameters among others MDA levels in cisplatin (CIS) induced toxicity in the liver and kidney tissues. MDA levels were the same for both liver and kidney control group and *p*-CA-treated group. However, significant effects of *p*-CA treatment were observed compared to the CIS-treated group. MDA content was over 1.5-fold lower in the *p*-CA-treated group compared to the CIS-treated group. The authors concluded that *p*-CA protects the liver and kidneys from CIS-induced oxidative damage in the model experiment. Other authors investigated the protective effects of CFA on the erythrocyte membrane. The results showed about 60% decrease in MDA production in the presence of 0.27 mmol/L CFA. However, the inhibitory effect of the acid was dependent on the concentration used [122]. The same trend was observed when studying the effect of RA on MDA production in low density lipoproteins (LDL). RA significantly inhibited the MDA production in LDL, but also in this case the inhibitory effect was dose-dependent [79]. Reducing effect of ChA on MDA production in hepatocyte models and larval zebrafish model was reported in [123]. In other studies on comparing the effects of ChA and CFA on lecithin liposome peroxidation it was found that ChA at concentration of 100 µmol/L inhibited liposome peroxidation by 55.6%, which was 2.81-fold higher than that of CFA. The ability for inhibiting liposome peroxidation by both studied acids was also dose-dependent [124].

### 3.7. Nitric Oxide Radical Scavenging Assay

The assay is based on the principle that sodium nitroprusside in aqueous solution at physiological pH spontaneously generates nitric oxide, which interacts with oxygen to produce nitrite ions that can be estimated using Griess reagent at 540 nm. The NO radical scavenging activity is often expressed as IC_50_ value (Table 3). *p*-CA (IC_50_ = 17 µmol/L) exhibits lower inhibition lipopolysaccharide-stimulated NO production in mouse macrophage like cells (RAW 264.7 cells) compared to CFA (IC_50_ = 0.5 µmol/L) [118]. They also refer biological activities of *p*-CA and CFA to the enthalpy of O-H bond dissociation energy (BDA). The IC_50_ value reported for RA was 43.49 µmol/L. RA showed 3.4× higher inhibition of nitric oxide radical production than Trolox [96].

The review of literature on the antioxidant capacity of NCA showed that information on RA, CFA and *p*-CA are more available, while on BA, CinA and ChA are very scarce. There are only a few papers in which antioxidant capacity of more than two out of six carboxylic acids described in this work were tested under the same experimental conditions [70,82,85,95,115,116]. Values obtained for four reviewed compounds are reported only in one paper [85]. Comparison of the results obtained using the same experimental protocol could also lead to misleading conclusions, because in the cited papers different concentrations of antioxidants were used. In many papers it was reported that increasing concentration of the tested compounds resulted in their higher reduction activity [84,120,124]. Moreover, the results of analysis could be influenced by the reaction time and the temperature. For instance, ABTS radical scavenging activity of CFA (RSA = 47.98%) obtained by Choi et al. [92] after 2 min of incubation was lower than the RSA value equal 92.9% obtained by Gülcin [76] after 30 min of incubation. The antioxidant activity of *p*-CA and CFA obtained by CUPRAC assay at room temperature [116] was higher than that achieved at enhanced (50 °C) temperature [66]. Moreover, different values were reported for the antioxidant activity of *p*-CA and CFA estimated by using the same experimental protocols of ABTS and DPPH [91,99] and by FRAP and CUPRAC methods [84]. It is obvious that the lack of standardized analytical methods for examination of antioxidant capacity and clear and comparable ways of expressing measurement results leads to the inconsistency of the reported data.

However, information gathered in this review allows to describe general trends and categorize the antioxidant activity of the tested compounds. Antioxidant capacity among plant-derived antioxidants decreases in the following order: RA > CFA > *p*-CA > CinA > BA. The data presented for ChA are very scarce, but it could be placed between *p*-CA and CFA or CFA and RA (according to the results obtained by DPPH and ABTS methods). It could be concluded that cinnamic acid is more effective antioxidant than its benzoic counterpart. Strong antioxidant properties of CFA and *p*-CA are due to a phenolic hydroxyl group that reacts with oxidants and free radicals to form the resonance-stabilized phenoxyl radical, and to the presence of a propenoic side chain, whose conjugated double bond could, by resonance, have a stabilizing effect on the phenoxyl radical. However, CFA exhibit stronger antioxidant capacity compared to *p*-CA, that could be explained by the intramolecular hydrogen bonding in ortho substituted phenols [78]. RA, the dimer of CFA, exhibited the highest antioxidant capacity among hydroxycinnamic acids, because it possesses four phenolic hydroxyl groups whose presence correlate with high antioxidant activity. Moreover, it has a conjugated structure, further stabilizing the aryloxyl radicals produced during the course of RA oxidation [116]. Additionally, ChA possess four phenolic hydroxyl groups and conjugated structure (tartaric acid ester of two caffeic acids) that contribute for the extension of the delocalization of electrons, enhance the stability of the phenoxyl radical, and improve its radical scavenging ability. However, its antioxidant activity is lower comparing to RA.

## 4. Antimicrobial Properties of NCA

**Figure 2:**
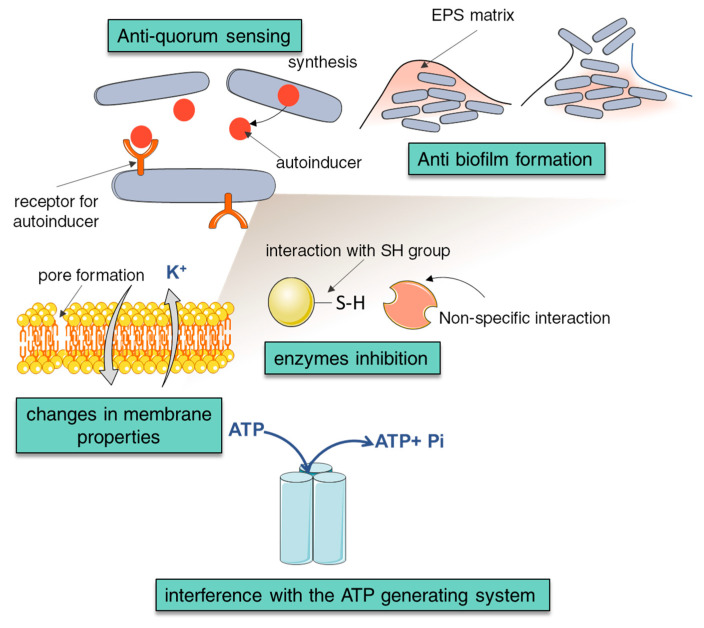
Selected mechanism of antimicrobial action of plant-derived carboxylic acids. [Based on [131]] Anti-quorum sensing. The quorum-sensing communication system can be inhibited in several different ways: Inhibition of autoinducers synthesis and transport, antagonist activity for autoinducers-receptors, and also direct reaction or inhibition of autoinducer activity. Changes in membrane properties. Phenolic acids (especially hydrophobic compounds) affect the properties of cell membranes (charge, permeability) through changes in hydrophobicity, reduction of negative surface charge, and the formation of pores in the membranes and leakage of intracellular components [135,136]. Anti-biofilm formation. Limiting the formation of biofilm by phenolic acids involves limiting cell adhesion to the surface and inhibiting biofilm maturation, indirectly through anti-quorum sensing action and inhibition of the expression of genes involved in biofilm formation [137,138]. Interference with the ATP generating system. Phenolic acids, by increasing the permeability of cell membranes, leak ions and partially inhibit the activity of ATPase [139] and other proteins, including enzymatic (enzymes inhibition). Acids with strong nucleophilic properties (e.g., CA) can donate an electron pair to electrophilic functional group of plasma membrane proteins and lipids leading to the membrane destabilization [140].

NCA have proven antimicrobial and antioxidant effects, which is why some of them have found application in food preservation, for example benzoic acid (E210), which occurs naturally in cranberry or cinnamon and propyl gallate (E310) synthesized from propanol and gallic acid. The antimicrobial potential of phenolic acids is associated with their chemical structure and depends on the number of hydroxyl (-OH) and methoxy (-OCH_3_) groups [125]. As antimicrobial compounds, they are often described as weak organic acids that diffuse across the cell membrane, acidify the cytoplasm and lead to cell death [126]. Therefore, pKa and lipophilicity are important parameters in the initial assessment of their bactericidal properties [125]. For example, caffeic acid as a hydroxycinnamic acid has a propene side chain, which makes it much less polar than, for example, protocatechuic acid. Therefore, caffeic acid as a less polar compound also exhibits higher lipophilicity, which may contribute to increase of cell membrane permeability [127] (Figure 2).

In studies conducted by Stojković et al. [128] it was observed that phenolic compounds such as caffeic acid, *p*-coumaric acid, and also rutin retain their antioxidant properties in situ in food. Among the mentioned acids, caffeic inhibited the growth to the greatest extent of *Staphylococcus aureus* developing in a food product. Analysing the antimicrobial activity of phenolic acids, it was found that hydroxycinnamic acids have comparable or better properties than hydroxybenzoic acids with the same number of hydroxyl groups. In addition, the antibacterial properties of hydroxybenzoic acids decrease as the number of -OH groups increases [128]. It was also observed that the longer side chains in the alkyl esters of caffeic acid showed better activity against Gram-positive bacteria, and the average chain length determined better activity against Gram-negative bacteria. The activity of the esters formed is directly related to lipophilicity, which affects the sensitivity of bacteria, the physicochemical properties of the bacteria and the integrity of cell membranes [129]. Generally, the structure of the cell wall of Gram-positive bacteria allows the penetration of hydrophobic molecules into the cell, while the membrane surrounding the wall of Gram-negative bacteria is virtually impermeable to them. Small hydrophilic compounds are able to penetrate the transmembrane channels; however, Gram-negative bacteria are usually more resistant to the action of antibiotics and hydrophobic toxins [130]. Higher lipophilicity enables the penetration of the acid molecule through the cell wall and membrane, where it disrupts the structure of individual layers of lipopolysaccharides, fatty acids and phospholipids and permeabilize them.

The highest MIC values were found for RA and the lowest for CFA and CinA (Table 4). Some studies; however, emphasize that the susceptibility of bacteria to phenolic compounds depends on the strain and the type of NCA [131]. For example, lactic acid bacteria have the ability to metabolize phenolic acids, and the produced metabolites have lower activity than the parent substrates [132]. The antibacterial effect of natural carboxylic acids can be due to many complex mechanisms. Most phenolic compounds disrupt the integrity and permeability of cell membranes, damaging their structure [131]. The literature also describes that some phenolic compounds (phenolic acids, flavonoids) have the ability to bind and intercalate with DNA structure, binding and blocking the activity of key proteins, e.g., DNA gyrase, kinase, dehydratase, helicase [133], and inhibition of topoisomerase, cytochrome c NADH reductase and ATP synthase [134]. Selected mechanism of antimicrobial action of natural carboxylic acids are presented in Figure 2. Interference with quorum sensing signalling pathways through interaction with receptors and molecules involved in intercellular communication is also not excluded. This is a particularly important feature in the fight against microorganisms that form a biofilms [131].

Compared with conventional antibiotics, NCA are not effective enough to classify them as antibiotics, but their high safety and availability in natural sources supports further research into their antimicrobial properties. Interestingly, there are more and more reports on synergistic effects of these compounds with antibiotics, especially in the fight against selected drug-resistant strains [141]. Kępa et al. [127] observed in their studies that caffeic acid enhances the action of known antibiotics against clinical strains of *Staphylococcus aureus* isolated from hard-healing wounds. A similar effect was observed for rosmarinic acid, which enhanced the effects of vancomycin, ofloxacin and amoxicillin against *S. aureus* against the drug-resistant MRSA strain. Interestingly, the lower concentrations of RA stimulated biofilm growth in a time- and concentration-dependent manner [142].

Hemaiswarya and Doble [143] observed that among the tested carboxylic acids (cinnamic, *p*-coumaric, caffeic, chlorogenic, ferulic, 3,4-dimethoxycinnamic, and 2,4,5-trimethoxycinnamic acid) used together with popular antibiotics (amikacin, ampicillin, ciprofloxacin, erythromycin and vancomycin), cinnamic, ferulic and *p*-coumaric acids showed the highest bactericidal activity against selected Gram-positive and Gram-negative bacteria strains. Interestingly, chlorogenic acid undergoing metabolic processes did not show sufficient activity in combination with antibiotics. Through the use of LIVE/DEAD^®^ BacLight™ tests, it has been observed that these acids damage cell membranes by increasing their permeability, which may partly explain the mechanism of their action [143].

## 5. Anticancer Activity of NCA

For many years, natural compounds of plant origin were the main source of oncological drugs, which over time underwent the necessary structural modifications to enhance their activity, bioavailability, and specificity [161,162]. Although experimental studies in cell or animal models have shown a positive relationship between the presence of phenolic compounds of natural origin and inhibiting the development of cancer cells, it is very difficult to extrapolate the results of these studies to cancer prevention or therapy in humans. One reason is that studies are often carried out at doses or concentrations far beyond those that can be achieved in patients. Available literature on the beneficial effects of polyphenols in human diets is based on in vitro or animal model experiments, but at concentrations far above those available in food sources. Mainly aglycons or conjugated forms are studied, most often without taking into account the active forms of metabolites, which does not provide complete information on the activity of these compounds in the body [163]. Nevertheless, studies on antioxidant (Table 3) and cytotoxic properties (Table 5) are very helpful in screening and preliminary assessment of their biological properties. The epidemiological data to date indicate a positive relationship between the intake of phenolic acid sources and the reduced incidence of some types of cancer.

In population studies, Russo et al. [187] observed a relationship between the consumption of phenolic acids and the risk of developing prostate cancer. It turned out that patients consumed less caffeic and ferulic acid than healthy men (CA: 2.28 Vs. 2.76 mg/day, ferulic acid: 2.80 Vs. 4.04 mg/day). This may suggest that a sufficient level of phenolic acids in the diet reduces the risk of developing prostate cancer. The effect of phenolic acid depends on the type of cancer. High and moderate coffee consumption, up to 5 cups a day, appears to be associated with significantly smaller sizes of oestrogen receptor alpha positive (ER+) invasive breast cancer, but no significant association with ER-type cancers was observed. In vitro tests have shown that exposure to caffeic acid is followed by a 50% reduction in MCF-7 cell proliferation and a 30% decrease in IGFIR levels. In addition, women with ER+ tumours drinking more than two cups of coffee a day during tamoxifen therapy showed reduced cancer recurrence compared to patients with low daily coffee intake [188]. Therapeutical potential of plant phenolic acids in prostate and breast cancer was also described, inter alia, in [189,190,191] and in recent review published by Abotaleb et al. [192]. In addition, caffeic acid has the property of reducing the mutagenic potential of sodium azide or nitrofurylacrylic acid by 20–35% [193]. Chicoric acid had no antimutagenic effect. It also prevents chromosomal aberrations and doxorubicin-induced cardiotoxicity in rats [194,195]. The rosmarinic acid also has an interesting profile of anti-cancer properties. Zhang et al. [196] observed that RA induces apoptosis and inhibits the migration of ovarian cancer cells and modulates the expression of Malat-1–long non-coding RNA associated with, among other, tumour metastasis. Other in vivo studies in xenograft mice indicate that RA also inhibits the growth of such pancreatic cells, where it increases expression of miR-506 while inhibiting MMP2/16 and Ki-67 [197].

**Figure 3:**
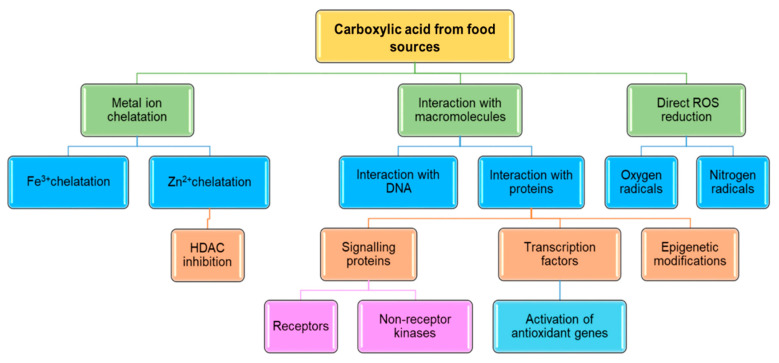
Selected molecular mechanisms of action of carboxylic acids from food sources. (HDAC—histone deacetylase).

Natural carboxylic acids, especially phenolic acids, are most often described in the context of antioxidant properties, but the mechanism of their action is very wide, not limited to reducing ROS. Small molecules can interact with receptors, nucleic acids and proteins that act as transcription factors and enzymes (Figure 3). Therefore, these compounds affect signal transduction pathways (e.g., redox sensitive Keap1/Nrf2/ARE system) and modifications of the chromatin structure, thus regulating gene expression, including those whose products are proteins involved in antioxidative defence and cell cycle regulation [198]. It has been proved that cinnamic acids, especially dihydroxycinnamic (caffeic) acid, have the ability to interact with HDAC2 (histone deacetylase 2), inhibiting its activity ex vivo and in vitro and inducing apoptosis of colon and cervical cancer cells [199]. It is worth noting that HDAC inhibitors are known potential anti-cancer drugs, among which there are inhibitors with a high affinity for zinc ions located in active deacetylase centres (e.g., hydroxamic acid and compounds having a benzamide group [200,201]). Due to the fact that carboxylic acid derivatives, also phenolic acids, possess chelating properties of metal ions (also Zn^2+^), they are potential HDAC inhibitors, which was confirmed by in silico and in vitro tests [202].

In the prevention of colon cancer, a large role is played by the products of the metabolism of the intestinal microbiome, which in addition to butyric acid and short-chain fatty acids are phenolic acids, e.g., *trans*-cinnamic acid formed in the process of deamination of phenylalanine [203]. The products of microbiome metabolism often have much better health-promoting properties than parent compounds, and in most cases biotransformation by microflora is necessary to ensure the bioavailability of phenolic acids. Only part of the phenolic compounds consumed in the form of glycosides is hydrolysed and absorbed in the small intestine. Ultimately, they undergo metabolism in the liver to conjugate forms—sulphates or glucuronides. Non-absorbed glycosides pass into the colon, where they are metabolized by the intestinal microbiome (e.g., flavonoids combined with rhamnose are hydrolysed by α-rhamnosidases produced by *Bifidobacterium dentium* [204,205]). Hence the final effect caused by phenolic acids in the body will depend on the content of phenolic compounds in food, their chemical form and the composition of the intestinal microflora. Although the activity of these acids is low compared to conventional inhibitors, this result indicates that the metabolic product of microorganisms inhabiting the gastrointestinal tract has antitumor potential. In studies published by Zhu et al. [206], it was noted that the administration of *trans*-cinnamic acid to rodents at a concentration of 1 and 1.5 mmol/kg body weight inhibited the growth of colon cancer xenografts, and the mechanism of action of this compound was partly due to HDAC inhibition in cancer cells. Phenolic acids also have direct antioxidant potential, protecting, among others before lipid peroxidation building biological membranes. Cancer cells are often characterized by elevated levels of ROS compared to healthy cells, but due to the inadequate bioavailability of phenolic compounds, their antioxidant intra-systemic activity is controversial.

Studies of Zambonin et al. [207] showed that phenolic acids (caffeic, syringic, and protocatechuic) reduce ROS and act antiproliferative and proapoptotic in leukaemia (HEL) cell lines, without causing any (antioxidant and toxic) effect on healthy cells (HUVEC). According to Wang and Yi [208] there are two opposing cancer therapy strategies based on the redox status of cancer cells. On the one hand, antioxidant therapy can effectively inhibit cell proliferation and neovascularization, which is a process in which free radicals participate, and also prevent the accumulation of mutations leading to genomic instability. At the same time, pro-oxidative therapy aimed at sufficiently increasing the concentration of free radicals in cancer cells may be a signal of initiation of apoptosis [209]. The biological properties of phenolic phytochemicals depend; however, on the amount of metabolized compound and the concentration obtained in the body. Ferulic and caffeic acids-the most common phenolic acids, after absorption undergo intensive metabolic processes, and some of these metabolites still retain strong antioxidant properties in vivo. In human plasma, both acids occur almost exclusively as conjugated forms—glucoronates and sulphates, and in addition synergistic effects in the presence of other products of metabolic processes are not excluded [210]. These compounds can also act indirectly on the reduction of free radicals by stimulating the synthesis of antioxidant enzymes—SOD (superoxide dismutase), CAT (catalase), and GPx (glutathione peroxidase) [211,212].

## 6. Structure Elements and Biological Activity

The biological activity of natural carboxylic acids depends on many physicochemical descriptors such as HOMO, LUMO energy, polarizability, size and shape of the molecule, dipole moment, solubility, hydrophilicity, lipophilicity, and presence of rotating bonds. The value of these descriptors affects the type and strength of phenolic acid interactions with various biological macromolecules such as cell membranes, cell walls, intracellular and membrane proteins, and DNA. Determining which descriptors are responsible for the expected biological effect of a chemical compound is very complex and may differ from physiological conditions, a cell line, or a bacterial strain. In addition to the widely discussed effects of phenolic acids on the redox balance of human cells, they affect both ROS-dependent and independent cell signalling pathways [192]. The QSAR (quantitative structure–activity relationship) analysis carried out by Uesawa et al. [213] showed that tumour specificity for phenolic cinnamic acid esters is correlated with the shape, size, and ionization potential (IP) of the molecules. Replacing one hydroxyl group with a methoxy group reduces cytotoxic activity several times [214]. The phenomenon that methylation of hydroxyl groups can improve antioxidant properties, but significantly reduce the cytotoxic potential was earlier reported by Fiuza et al. [214]. Enhancing phenolic acid activity, that was observed by Li et al. [215], may be the effect of formation of esters that exhibit visible cytotoxic activity as opposed to their respective non-toxic phenolic acids (IC_50_ > 100 µmol/L). On the other hand, among compounds of the same alkyl chain length, trihydroxylated esters showed a higher antiproliferative and cytotoxic effect than those having two –OH groups [214]. In several experiments, it was noted that phenolic compounds affect the fluidity of phospholipid membranes, induce their aggregation and rigidity, and this feature is associated with the number of hydrophilic side chains. Polar hydroxide groups are able to form hydrogen bonds with head group of membrane phospholipids, mediate in phospholipid aggregation, thus causing a decrease in membrane area and rendering membrane more rigid. It turns out that opening the aromatic C ring to olefin bonds, present, for example, in resveratrol, allows for the insertion of such compounds deeply into the hydrophobic interior of lipid bilayers and makes the cell membrane more fluid [216]. For this reason, the presence of –OH groups is thought to be a key in interacting with cell membranes. Phenolic hydroxyl groups play the role of hydrogen bond donors, while oxygen atoms in phospholipids may act as acceptors [217].

The presence of functional groups determines the distribution of electron charge in a molecule, and therefore its ability to bind to the surface of proteins or cell membranes. At physiological pH, mammalian cells have a negative surface charge determined by the presence of carboxyl, phosphate and ammonium groups on the outside of the cell. The electrical properties of biological membranes can be modified by interaction with different compounds, including phenolic acids. The location and permeability of polyphenols through membranes depends on the pH and charge. For example, using model lipid membranes, it was shown that at a lower pH, *p*-coumaric acid has a lower deprotonation state and penetrates deeper into the cell, so this is a feature conditioning its biological efficiency [218].

Slightly different physicochemical features will characterize compounds with high affinity for proteins or DNA. Comparing the ability of phenolic compounds to bind to bovine serum albumin (BSA), it was observed that chlorogenic acid has the highest affinity, whereas caffeic acid has lower affinity. The binding affinity of studied compounds decreases in the order: chlorogenic > caffeic > *m*-coumaric ≥ *p*-coumaric > ferulic > synaptic acid. The authors suggest that these molecules bind to the protein mainly through hydrophobic interactions and hydrogen bonds. Modelling has established that the phenyl group is in direct contact with the protein binding pocket, while the carboxyl group is oriented towards the solvent [219].

As with cytotoxicity to human cells that are strictly dependent on the cell line and physiological conditions, the antimicrobial effect of polyphenols is specific for the bacterial species and its environment. Generally, phenolic compounds with higher lipophilicity show better bactericidal activity. Esterification (chlorogenic acid as an ester of caffeic and quinic acid) or glycosylation reduce the compound’s lipophilicity, and thus lower its efficacy against *S. Enteritidis* or *E. coli* strains. The relatively low susceptibility of *P. aeruginosa* to polyphenols compared to other bacterial strains may result from the presence of an impermeable outer membrane as well as the existence of multi-drug resistance pumps. Some phenolic compounds such as epigallocatechin gallate (EGCG) having the ability to inhibit efflux pomps systems are highly effective and may increase the sensitivity of pathogens to conventional antibiotics [220]. It seems that the dominant mechanism of phenolic activity is the interactions between polyphenols and bacterial cells surface, which is why hydrophobicity is a property of the molecule that will largely determine its effectiveness [131].

## 7. Conclusions

In this review, we analysed the physicochemical and biological properties of the natural carboxylic acids series naturally occurring in aromatic plants and spices—benzoic acid, cinnamic acid, and its hydroxyl derivatives (*p*-coumaric and caffeic acids)—and selected esters (rosmarinic and chicoric acids) and compared the effect of structural differences on their antioxidant, antimicrobial, and cytotoxic activity. Cinnamic acid is a precursor for the synthesis of various derivatives having hydroxyl groups as well as different esters. An example of such compounds are chicoric acid (a derivative of caffeic and tartaric acid) and rosmarinic acid (an ester of caffeic acid and 3,4-dihydroxyphenyllactic acid). An additional hydroxyl groups and two carboxyl groups of the tartaric moiety present in the structure of chicoric acid may improve its solubility and chelating capacity. Therefore, chicoric acid is relatively soluble in the cellular environment.

The literature analysis has shown that rosmarinic acid has the highest antioxidant activity and this property decreases in the following order: rosmarinic > caffeic acid > *p*-coumaric acid > cinnamic acid > benzoic acid. The small number of papers on chicoric acid makes difficult to compare it with other compounds, but its structure indicates that it could possess higher antioxidant properties than *p*-coumaric acid. The log *p* values for chicoric acid (Table 2) display its low lipophilicity. There is a clear relationship between the number of hydroxyl groups and antioxidant properties, and the presence of conjugated structures enable better stabilization of the phenoxy radical formed. In addition, the mechanism of the redox reaction of rosmarinic acid is due to the reversible oxidation of catechol groups, depending on the pH.

We did not include other hydroxybenzoic acids in our review; however, a small size of hydroxybenzoic acids positively affects their diffusion properties and general antioxidant capacity. Many studies show that hydroxycinnamic acid has a higher antioxidant activity than hydroxybenzoic acid, which may be related to the electron donating capacity of carboxyl groups [221]. The donor group increases the electron cloud density of the benzene ring, reduces the dissociation energy of the phenolic hydroxyl bond, and increases its ability to scavenge free radicals. Therefore, carboxylic acid groups affect the antioxidant activity of phenolic acids according to their electron-donating ability in the following order: -CH_2_COOH > -CH = CHCOOH > -COOH.

In the case of antimicrobial properties, structure-activity relationships are not easy to observe as they closely depend on the experimental conditions and the microbial strain. There are a lot of mechanisms in the case of antibacterial action: From lowering the pH of the cytosol, chelating essential transition metal ions, disrupting quorum-sensing intercellular communication, to disturbing the integrity of cell membranes and efflux of cytoplasmic constituents and release of intracellular K^+^ ions (Figure 2). Phenolic acids also inhibit the activity of bacterial enzymes, disrupting their metabolism and depriving the substrates necessary for growth. In the case of hydroxycinnamic acids, a higher ion leakage and a greater influx of protons into the cells is observed than in hydroxybenzoic acids [222]. Additionally, the analysed series of compounds meets Lipiński’s rules, which proves their functional potential as drugs and antimicrobial agents.

Current reports on the anti-cancer properties of phenolic acids focus on explaining the mechanism of their action. It was found, inter alia, that acids, in addition to their anti-radical activity, can bind to specific cellular proteins, acting as inhibitors (e.g., inhibition of MAPK4 by rosmarinic acid in cancer cells [223] or inhibition of phosphatase in pathogenic bacteria YopH by chicoric acid on the basis of allosteric inhibition [224]). Some processes are closely related and ROS-dependent signalling pathways are particularly sensitive to the presence of antioxidants as phenolic acids [225]. It seems that hydroxyl groups are particularly important, not only in the reduction of free radicals, but also in intermolecular interactions and shaping the cytotoxic potential, while the carboxyl group participates in the chelation of endogenous transition metal ions acting as, for example, HDAC inhibitors [200].

The above considerations could be useful for the design and synthesis of compounds possessing desired biological activity. Such compounds can be effectively employed as natural preservatives in various food products. The future research on the structure-activity relationship of complexes of NCA with different metal ions should be also undertaken, as no in-depth study is available in the literature so far. Complexation of ligands with metals, through the changes in the distribution of a charge in a ligand and the lipophilic character of these compounds, as well as alteration in the structural conformation of metal complexes, may positively influence their biological activity and affect their fate in the organisms. The studies conducted in the biological systems are extremely important, as other constituents present is biological environment may influence the stability and activity of such compounds.

## Figures and Tables

**Table 1:** Concentration of NCA in different aromatic plants (herbs and spices).

Plant	Total Phenols		BA		CinA		*p*-CA		CFA		RA		ChA	
	[mg GAE/g DW]		[mg/g DW]											
Cinnamon —*Cinnamomum verum J.Presl*—*Lauraceae*	5.82 ± 0.44	[21]	0.461	[15]	_		0.0022	[21]	0.153 ^e^	[19]	0.00073	[21]	_	
			54.40–391.99 *	[25]					0.00045	[21]				
—*Cinnamomum cassia* (L.) *J.Presl*—*Lauraceae*	_		_		0.01–1.91	[14]	_		_		_		_	
Rosemary —*Rosmarinus officinalis* L.—*Lamiaceae*	5.02 ± 0.43	[21]	n.s.	[25]	_		0.0056	[21]	L: 41.42 ± 51 *^,a^ R: 112.40 ± 51 *	[26]	L: 15.14 ± 19 *^,a^ R: 156.61 ± 65 *^,a^	[26]	_	
									0.401 ^e^	[19]	12.86 ^e^	[19]		
									0.0126	[21]	0.157	[21]		
									4.06	[18]	328 ± 16.9 **	[27]		
									29.5 ± 1.2 **	[27]				
Thyme —*Thymus vulgaris* L.—*Lamiaceae*	3.36 ± 0.14	[21]	0.015–0.050	[15]	_		0.0027	[21]	L: 179.65 ± 8 *^,a^ R: 67.00 ± 4 *^,a^ F: 82.27 ± 12 *	[26]	L: 392.21 ± 1 *^,a^ R: 104.20 ± 12 *^,a^ F: 104.20 ± 3 *	[26]	_	
			n.s.	[25]					0.548 ^e^	[19]	6.81 ^e^	[19]		
									0.0066	[21]	0.084	[21]		
									5.17	[18]	918 ± 27.5 **	[27]		
									117 ± 10.4 **	[27]				
Oregano —*Origanum vulgare* L.—*Lamiaceae*	2.23 ± 0.18	[21]	≤LOQ	[25]	_		0.0049	[21]	0.500 ^e^	[19]	0.0520	[21]		
									0.0106	[21]	25.63 ^e^	[19]		
									0.500 ^e^	[19]				
									6.49	[18]				
	39.56 ± 0.42 ^b^ 9.73 ± 0.07 ^c^ 56.83 ± 1.65 ^d^ 22.12 ± 0.30 ^e^	[28]					0.109 ± 0.002 ^b^ 0.054 ± 0.002 ^c^ 0.365 ± 1.050 ^d^ 0.065 ± 0.003 ^e^	[28]	0.172 ± 0.010 ^b^ 0.258 ± 0.019 ^c^ 0.367 ± 0.008 ^d^ 0.180 ± 0.008 ^e^	[28]	7.599 ± 0.115 ^b^ 4.303 ± 0.113 ^c^ 19.269 ± 1.035 ^d^ 6.958 ± 0.071 ^e^	[28]	0.323 ± 0.830 ^b^ 0.160 ± 0.004 ^c^ 0.910 ± 0.040 ^d^ 0.355 ± 0.007 ^e^	[28]
—*Origanum acutidens* L.—*Lamiaceae*	_		_		_		0.068 ± 0.003 ^b^ 0.002 ± 0.001 ^c^ 0.267 ± 0.011 ^d^ ND ^e^	[28]	0.024 ± 0.002 ^b^ 0.478 ± 0.015 ^c^ 0.092 ± 0.003 ^d^ 0.096 ± 0.012 ^e^	[28]	0.392 ± 0.012 ^b^ 4.858 ± 0.435 ^c^ 6.951 ± 0.539 ^d^ 0.525 ± 0.004 ^e^	[28]	ND^b^ 0.003 ± 0.001 ^c^ ND ^d^ ND ^e^	[28]
Basil —*Ocimum basilicum* L.—*Lamiaceae*	L: ≤ LOQ –45.69 * S: ≤ LOQ *	[25]	_		_		0.204 ^e^	[19]	10.86 ^e^	[19]				
fresh	L: 6.516 R: 2.234	[24]	_		_		_		_		L: 1.386 R: 0.376	[24]	L: 0.370 R: 0.075	[24]
dry	4.236	[24]	_		_		_		_		0.557	[24]	0.107	[24]
Caraway —*Carum carvi* L.—*Apiaceae*	_		≤LOQ *	[25]	_		0.0106	[29]	0.164	[19]	_		_	
									0.010	[29]				
Turmeric —*Curcuma longa* L.—*Zingiberaceae*	_		0.003–0.005	[15]	_		0.0011	[29]	_		_		_	
			≤LOQ–71.47 *	[25]										
Marjoram —*Origanum majorana* L.—*Lamiaceae*	_		_		_		_		0.0002	[29]	_		_	
—*Origanum x majoricum*—*Lamiaceae*	_		_				104 ± 2.7 **	[27]	1546 ± 32.9 **	[27]	_		_	
Pepper —*Piper nigrum* L. —*Piperaceae*	_		0.003–0.005	[15]	_		_		_		_		_	
			≤LOQ–25.90 *	[25]										
Cumin —*Cuminum cyminum* L.—*Apiaceae*	4.98 ± 0.31	[21]	_		_		0.00074	[21]	0.0031	[21]	0.0033	[21]	_	
Bay —*Laurus nobilis* L.—*Lauraceae*	1.12 ± 0.08	[21]	_		_		0.0096	[21]	0.0004	[21]	0.00039	[21]	_	
Sage —*Salvia officinalis* L.—*Lamiaceae*	_				_		_		1.215 ^e^	[19]	21.86 ^e^	[19]	_	
									2.96	[18]	1178 ± 10.1 **	[27]		
									74.2 ± 3.5 **	[27]				
Melisa —*Melisa officinalis* L.—*Lamiaceae*	_		_		_		_		8.580	[18]	_		_	
Mint —*Mentha canadensis* L.—*Lamiaceae*	_		_		_		_		0.271 ^e^	[19]	19.085 ^e^	[19]	_	
*Echinacea purpurea* L.—*Asteraceae* fresh	4.441	[24]	_		_		_		_		_		2.423	[24]
extract	1640 *	[24]	_		_		_		_		_		323 *	[24]
Nutmeg —*Myristica fragrans Houtt.*—*Myristicaceae*	_		0.217	[15]	_		_		0.163 ^e^	[19]	_		_	
			≤LOQ *	[25]										
Parsley —*Petroselinum crispum* L.—*Apiaceae*	_		<0.001	[15]	_		_		1.037 ^e^	[19]	_		_	
			≤LOQ–4.11 *	[25]										
White pepper —*Piper nigrum* L.—*Piperaceae*	_		0.001–0.003	[15]	_		_		_		_		_	
			≤LOQ	[25]										

* results expressed as mg/L; ** results expressed as mg/kg of fresh weight (FW); ≤LOQ—below the limit of quantification; n.s.—no significant; n.d.—no data; L—leaf; R—stem; F—flower; S—seed; DW—dry weigh. Extract in: ^a^ oil in water emulsions; ^b^ water; ^c^ ethyl acetate; ^d^ methanol; ^e^ ethanol.

**Table 2:** The basic physicochemical parameters of NCA.

Acid	BA			CinA			*p*-CA			CFA			RA			ChA		
M_w_	122.12		[30]	148.16		[30]	164.16		[30]	180.16		[30]	360.31		[30]	474.37		[30]
pK_a_	4.19	^1^	[31]	4.44	^1^	[32,33]	4.64	^1^	[34]	4.36	^1^	[35]	3.57	^1^	[36]			
	4.21	^1^	[33]				4.70	^1^	[37]	4.41	^1^	[38]	3.62	^1^	[39]			
							9.15	^2^	[40]	4.49	^1^	[41]						
							9.50	^2^	[34]	8.48	^2^	[35]						
										8.72	^2^	[41]						
										8.85	^2^	[40]						
										>10	^3^	[40]						
										11.17	^3^	[35]						
										11.38	^3^	[35]						
pK_a_ calculated	4.2	^1^	[42]	4.3	^1^	[42]	4.6	^1^	[42]	4.6	^1^	[42]	2.8	^1^	[42]	2.72	^1^	[43]
							4.65	^1^	[44]	9.8	^2^	[42]	2.78	^1^	[45]			
							10.2	^2^	[42]	12.8	^3^	[42]	9.3	^2^	[42]			
							9.92	^2^	[44]				9.33	^2^	[45]			
													9.8	^3^	[42]			
													9.77	^3^	[45]			
													12.3	^4^	[42]			
													12.33	^4^	[45]			
													12.6	^5^	[42]			
													12.65	^5^	[45]			
Log *p*	1.87		[46]	2.13		[46,47]	1.46		[48]	1.15		[48]	1.60		[49]	0.72		[49]
	2.03		[50]	2.08		[51]	1.79		[47]				1.63		[52]	1.23		[43]
Log *p* calculated	1.89		[42]	2.41		[42]	1.88		[42]	1.42		[42]	1.70		[42]	3.02		[43]
																3.48		[43]
HBA	2		[53]	2		[54]	3		[54]	4		[54,55]	8		[52]	11		[56]
													4		[49]	10		[43]
																8		[49]
HBD	1		[53]	1		[54]	2		[54]	3		[54,55]	5		[52]	6		[56]
													4		[49]	4		[49,43]

^1^—pK_a1_, ^2^—pK_a2_, ^3^—pK_a3_, ^4^—pK_a4_, ^5^—pK_a5_; HBA—H-bond acceptor, HBD—H-bond donor.

**Table 3:** Antioxidant activity of NCA.

Method	BA		CinA		*p*-CA		CFA		RA		ChA	
DPPH, IC_50_ [µmol/L]	n.d.		>160 (C_DPPH_ = 150 µmol/L; t = 30 min)	[74]	3.96 ± 0.06 (C_DPPH_ = 50 µmol/L; t = 30 min)	[75]	0.17 (C_DPPH_ = 304.3 µmol/L; t = 30 min)	[77]	0.5 ± 0.03 (C_DPPH_ = 1000 μmol/L, t = 30 min)	[79]	8.6 ± 0.9 (C_DPPH_ = 1000 µmol/L; t = 15 min)	[81]
			>250 (C_DPPH_ = 250 µmol/L; t = 30 min)	[82]	6.65 (C_DPPH_ = n.d.; t = n.d.)	[83]	0.97 (C_DPPH_ = n.d.; t = n.d.)	[83]	1.83 ± 0.08 (C_DPPH_ = 100 μmol/L, t = 30 min)	[96]	140 (C_DPPH_ = 553 µmol/L; t = 15 min)	[97]
			n.s. (C_DPPH_ = 101 µmol/L; t = 30 min)	[87]	163.1 (C_DPPH_ = 304.3 µmol/L; t = 30 min)	[77]	1.55 ± 0.22 (C_DPPH_ = 500 µmol/L; t = 30 min)	[75]	4.19 ± 0.19 (C_DPPH_ = 100 μmol/L, t = 30 min)	[98]		
					>250 (C_DPPH_ = 250 µmol/L; t = 30 min)	[82]	4.72 (C_DPPH_ = 101 µmol/L; t = 30 min)	[5]	6.33 (C_DPPH_ = 55 μmol/L, t = 30 min)	[94]		
					7817 ± 77 (C_DPPH_ = 75 µmol/L; t = 30 min)	[91]	13.3 (C_DPPH_ = 101 µmol/L; t = 30 min)	[99]	9.16 ± 0.31 (C_DPPH_ = 0.254 µmol/L, t = n.d.)	[100]		
					12,800 ± 100 (C_DPPH_ = 50 µmol/L; t = 30 min)	[95]	21.7 ± 0.2 (C_DPPH_ = 6.85 µmol/L, t = 1–45 min)	[93]	72.3 ± 3.3 (C_DPPH_ = 200 µmol/L, t = 30 min)	[101]		
					n.s.	[62]	32.2 (C_DPPH_ = 100 µmol/L; t = n.d.)	[102]	230 (C_DPPH_ = 101 µmol/L, t = 15 min)	[103]		
							35.2 ± 2.1 (C_DPPH_ = 75 µmol/L; t = 30 min)	[91]	381 ± 11 (C_DPPH_ = 1000 µmol/L, t = 30 min)	[104]		
							50.0 (C_DPPH_ = 355 µmol/L; t = 30 min)	[105]	1210 (C_DPPH_ = 100 μmol/L, t = 30 min)	[106]		
							155.3 (C_DPPH_ = 250 µmol/L; t = 30 min	[82]				
							1110 ± 10 (C_DPPH_ = 63.4 µmol/L; t = 2 min)	[62]				
DPPH radical scavenging activity [%]	0 (C_BA_ = 1000 µmol/L, C_DPPH_ = 60 µmol/L; t = 30 min)	[70]	0.5 (C_CinA_ = 169 µmol/L; C_DPPH_ = 100 µmol/L; t = 20 min)	[85]	3.6 (C*_p_*_-CA_ = 152 µmol/L; C_DPPH_ = 100 µmol/L; t = 20 min)	[85]	28.5 (C_CFA_ = 167 µmol/L; C_DPPH_ = 100 µmol/L; t = 30 min)	[84]	88.4 (C_RA_ = 69 µmol/L; C_DPPH_ = 100 µmol/L; t = 20 min)	[85]	55.6 (C_ChA_ = 25 µmol/L; C_DPPH_ = 200 µmol/L; methanol, λ = 517 nm, t = 30 min	[80]
	~4 (C_BA_ = 15 µmol/L; C_DPPH_ = 60 µmol/L; t = 30 min)	[71]	2.06 — 3.25 (C_CinA_ = 17—135 µmol/L; C_DPPH_ = 1000 µmol/L; t = 30 min)	[107]	5.3 ± 0.50 (C*_p_*_-CA_ = 167 µmol/L, C_DPPH_ = 80 µmol/L; t = 10 min;)	[84]	46.1 — 75.8 (C_CFA_ = 1—5 µmol/L, C_DPPH_ = n.d.; t = n.d.)	[83]				
			~35(C_CinA_ = 1000 µmol/L, C_DPPH_ = 60 µmol/L; t = 30 min)	[70]	~27 (C*_p_*_-CA_ = 1000 µmol/L, C_DPPH_ = 60 µmol/L; t = 30 min)	[70]	47.8 (C_CFA_ = 50 µmol/L; C_DPPH_ = 0.25 µmol/L; t = 30 min)	[108]				
			60.3 —62.5 (C_CinA_ = 675 i 1350 µmol/L; C_DPPH_ = 1000 µmol/L; t = 30 and 60 min)	[72]	30.1—43.9 (C*_p_*_-CA_ = 1—5 µmol/L, C_DPPH_ = n.d.; t = n.d.)	[83]	17.5 ± 0.2 (C_ABTS_ = 2.45 mmol/L; t = 15 min)	[93]				
					43.9 ± 9.2 (C*_p_*_-CA_ = 5000 µmol/L; C_DPPH_ = n.d.; t = n.d.)	[83]	100 ± 1 (C_ABTS_ = 7 mmol/L, t = 30 min)	[91]				
					55.6 (C*_p_*_-CA_ = 167 µmol/L C_DPPH_ = 100 µmol/L; t = 30 min;)	[73]	1010 ± 0 (C_ABTS_ = 30 µmol/L; t = 2 min)	[62]				
					n.s.	[62]	51.5 (C_CFA_ = 20 µmol/L; C_DPPH_ = 0.1 µmol/L; t = 30 min)	[109]				
							76.6 (C_CFA_ = 25 µmol/L; C_DPPH_ = 100 µmol/L; t = 20 min	[85]				
							93.9 (C_CFA_ = 111 µmol/L, C_DPPH_ = 100 µmol/L, t = 30 min)	[76]				
ABTS, IC_50_ [µmol/L]	n.d.		n.s.	[82] [87] [110] [111]	50.0 ± 3.3 (C_ABTS_ = 7 mmol/L, t = 30 min)	[91]	10.9 (C_ABTS_ = 7 mmol/L, t = 6 min)	[99]	2.91 (C_ABTS_ = 7 mmol/L, t = 6 min)	[94]	n.d.	
ABTS radical scavenging activity [%]	0 (C_BA_ = 5 mmol/L; C_ABTS_ = 0.15 mmol/L; t = n.d.)		n.d.		51.7±0.41 (C*_p_*_-CA_ = 183 µmol/L; C_ABTS_ = 6 mmol/L; t = 10 min;)	[84]	32.1% (C_CFA_ = 167 µmol/L; C_ABTS_ = 6 mmol/L; t = 2 min)	[84]	n.d.		49.1% (C_ChA_ = 105 µmol/L; C_ABTS_ = 7 mmol/L; t = 6 min)	[80]
	<1% (C_BA_ = 0.01 mmol/L; C_ABTS_ = 2.45-mmol/L; t = 7 min)	[90]					47.98% (C_CFA_ = 111 µmol/L; C_ABTS_ = 6 mmol/L, t = 2 min)	[92]				
							92.9% (C_CFA_ = 111 µmol/L; C_ABTS_ = 7 mmol/L; t = 30 min)	[76]				
FRAP [μmol Fe^2+^/L]	n.d.		n.d.		n.d.		42 (C = 10 μmol/L; λ = 593 nm, t = 30 min)	[80]	37.813 (λ = 596 nm, t = 15 min)	[104]	82 (C = 10 μmol/L; λ = 593 nm, t = 30 min)	[80]
							180 (C = 500 µmol/L; λ = 593 nm, t = 30 min)	[80]			145 (C = 500 µmol/L; λ = 593 nm, t = 30 min)	[80]
FRAP, IC_50_ [µmol/L]	n.d.		>200,000	[95]	420	[95]	60	[112]	19.6	[113]	n.d.	
							120	[95]				
FRAP [%]	n.d.		n.d.		4.6 (C = 167 µmol/L)	[84]	30.8 (C = 167 µmol/L)	[84]	n.d.		n.d.	
CUPRAC [µmol Trolox equ./mg of pure compound]	n.d.		n.d.		0.55	[66]	2.6	[114]	4.88	[114]		
					0.55	[115]	2.89	[66]	4.95	[116]		
							2.89	[115]	5.65	[115]		
					1.12	[116]	3.4	[116]			n.d.	
CUPRAC [%]	n.d.		n.d.		1.37 (C = 167 µmol/L)	[84]	3.3 (C = 167 µmol/L)	[84]	n.d.		n.d.	
ORAC [µmol Trolox equ./mg of pure compound]	n.d.		n.d.		1.67	[62]	2.75	[62]	n.d.		n.d.	
					4.51	[117]	6.63	[117]				
Nitric Oxide Radical Scavenging Assay, IC_50_ [µmol/L]	n.d.		n.d.		17	[118]	0.5	[118]	43.49	[96]	n.d.	

n.d.—no data, n.s.—not significant.

**Table 4:** Antimicrobial effects of NCA on selected microbial strains.

Compound	Relation	Value [mmol/L]	Standard	Microorganism Strain	Source
BA	>=	0.52	MIC	*Staphylococcus aureus*	[144]
	>	1.05	MIC	*Staphylococcus aureus*	[144]
	=	6.55	MIC	*Saccharomyces cerevisiae*	[145]
	=	6.55	MIC	*Saccharomyces cerevisiae*	[145]
	=	6.55	MIC	*Saccharomyces cerevisiae*	[145]
	=	6.55	MIC	*Phellinus tremulae*	[146]
	>	13.10	MIC	*Saccharomyces cerevisiae*	[145]
	>	13.10	MIC	*Saccharomyces cerevisiae*	[145]
	=	0.33	MIC	*Mycobacterium tuberculosis*	[147]
	=	5.00	MIC	*Cochliobolus lunatus*	[148]
	=	5.00	MIC	*Lasiodiplodia theobromae*	[146]
	=	5.00	MIC	*Neofusicoccum ribis*	[146]
	=	5.00	MIC	*Diplodia seriata*	[146]
	=	5.00	MIC	*Botryosphaeria dothidea*	[146]
CinA	=	3.37	MIC	*Aspergillus parasiticus*	[149]
	>	0.86	MIC	*Streptococcus pyogenes*	[150]
	>	0.86	MIC	*Staphylococcus aureus*	[150]
	=	0.27	MIC	*Mycobacterium tuberculosis H37Rv*	[151]
	=	1.68	MIC	*Aspergillus niger*	[149]
	=	3.37	MIC	*Staphylococcus aureus*	[149]
	>	0.86	MIC	*Staphylococcus epidermidis*	[150]
	=	6.75	MIC	*Klebsiella pneumoniae*	[149]
	=	6.75	MIC	*Bacillus subtilis*	[149]
	>	0.86	MIC	*Pseudomonas aeruginosa*	[150]
	>	0.86	MIC	*Staphylococcus aureus*	[150]
	=	0.42	MIC	*Trichophyton rubrum*	[149]
	>	0.86	MIC	*Escherichia coli*	[150]
	=	0.84	MIC	*Issatchenkia orientalis*	[149]
	=	6.75	MIC	*Burkholderia cepacia*	[149]
	=	6.75	MIC	*Micrococcus luteus*	[149]
	=	6.75	MIC	*Enterobacter cloacae*	[149]
	>	0.86	MIC	*Bacillus subtilis*	[150]
	>	0.60	MIC	*Mycobacterium smegmatis str. MC2 155*	[151]
	=	13.50	MIC	*Pseudomonas aeruginosa*	[149]
*p*-CA	>	0.60	MIC	*Mycobacterium smegmatis str. MC2 155*	[151]
	=	5.00	MIC	*Diplodia seriata*	[146]
	>	305	MIC	*Bacillus subtilis*	[152]
	=	0.24	MIC	*Mycobacterium tuberculosis H37Rv*	[151]
	=	5.00	MIC	*Neofusicoccum ribis*	[146]
	>	305	MIC	*Candida albicans*	[152]
	>	305	MIC	*Staphylococcus aureus*	[152]
	=	5.00	MIC	*Botryosphaeria dothidea*	[146]
	>	305	MIC	*Pseudomonas fluorescens*	[152]
	=	0.37	MIC	*Mycobacterium bovis BCG*	[151]
CFA	>	0.28	MIC	*Bacillus subtilis*	[152]
	>	0.28	MIC	*Staphylococcus aureus*	[152]
	=	0.71	MIC_50_	*Candida albicans*	[153]
	=	0.69	MIC	*Streptococcus pyogenes*	[154]
	=	0.69	MIC	*Staphylococcus aureus*	[154]
	>	0.30	IC_50_	*Saccharomyces cerevisiae*	[155]
	>	0.28	MIC	*Pseudomonas fluorescens*	[152]
	>	0.35	IC_50_	*Agaricus bisporus*	[156]
	=	1.47	MIC_50_	*Candida albicans*	[153]
	=	0.28	MIC_50_	*Candida albicans*	[153]
	>	0.28	MIC	*Candida albicans*	[152]
	=	0.71	MIC_50_	*Candida albicans*	[153]
RA	>	5556	MIC	*Aspergillus niger*	[157]
	=	11111	MIC	*Aspergillus niger*	[158]
	=	333	MBC	*Bacillus subtilis* subsp. *spizizenii*	[157]
	=	11111	MBC	*Bacillus subtilis* subsp. *spizizenii*	[158]
	=	11111	MIC	*Bacillus subtilis* subsp. *spizizenii*	[158]
	>	5556	MIC	*Candida albicans*	[157]
	=	5556	MIC	*Candida albicans*	[158]
	>	5556	MIC	*Escherichia coli*	[157]
	=	333	MBC	*Escherichia coli*	[157]
	>	5556	MIC	*Pseudomonas aeruginosa*	[157]
	=	333	MBC	*Pseudomonas aeruginosa*	[157]
	=	11111	MBC	*Pseudomonas aeruginosa*	[158]
	=	5556	MIC	*Pseudomonas aeruginosa*	[158]
	=	333	MIC	*Staphylococcus aureus*	[157]
	=	333	MBC	*Staphylococcus aureus*	[157]
	=	333	MBC	*Staphylococcus aureus*	[158]
	=	333	MIC	*Staphylococcus aureus*	[158]
	=	333	MIC	*Staphylococcus epidermidis*	[157]
	=	333	MBC	*Staphylococcus epidermidis*	[157]
	=	2778	MBC	*Staphylococcus epidermidis*	[158]
	=	333	MIC	*Staphylococcus epidermidis*	[158]
D-ChA	=	0.0039	Ki	*Clostridium botulinum* *	[159]
	=	0.0067	Ki	*Clostridium botulinum* #	[159]
	=	0.0016	Ki	*Clostridium botulinum* **	[159]
	=	0.0014	Ki	*Clostridium botulinum* ##	[159]
L-ChA	=	0.0158	Potency	*Bacillus anthracis str. A2012*	[160]

MIC—minimum inhibitory concentration, MIC_50_—minimum inhibitory concentration required to inhibit the growth of 50% of microorganisms, Ki—inhibitory constant, MBC—minimum bactericidal concentration, IC_50_—the half maximal inhibitory concentration. Uncompetitive inhibition of Clostridium botulinum: * full length BoNT/A light chain (1-448) using truncated SNAP 25 (141-206) peptide as substrate by LC/MS analysis, ** truncated BoNT/A light chain (1-425) using truncated SNAP 25 (141-206) peptide as substrate by LC/MS analysis, Competitive inhibition of Clostridium botulinum: # truncated BoNT/A light chain (1-425) using truncated SNAP 25 (141-206) peptide as substrate by LC/MS analysis, ## full length BoNT/A light chain (1-448) using truncated SNAP 25 (141-206) peptide as substrate by LC/MS analysis.

**Table 5:** Effects of NCA on selected cell lines in in vitro assays.

Compound	Relation	Value	Unit	Standard	Assay	Cell Line	Source
BA	>	10	µmol/L	IC_50_	Cytotoxicity against human cells after 48 h by SRB assay	BT-549	[164]
	>	10	µmol/L	IC_50_		A549	[164]
	>	10	µmol/L	IC_50_		SK-MEL-2	[164]
	>	50	µmol/L	IC_50_	Antineuroinflammatory activity in mouse BV2 cells assessed as inhibition of LPS-induced nitric oxide production after 24 h by Griess assay	BV-2	[164]
	>	10	µmol/L	IC_50_	Cytotoxicity against human SKOV3 cells after 48 h by SRB assay	SK-OV-3	[164]
CinA	=	64	µmol/L	IC_50_	Anticancer activity against human cells after 48 h by MTT assay	A-375	[165]
	=	108	µmol/L	IC_50_		MCF7	[165]
	=	91	µmol/L	IC_50_		ACHN	[165]
	=	87	µmol/L	IC_50_		A549	[165]
	=	114	µmol/L	IC_50_		HT-29	[165]
	>	100	µmol/L	IC_50_	Antineuroinflammatory activity in mouse BV2 cells assessed as inhibition of LPS-induced NO production after 24 h in presence of LPS by Griess assay	BV-2	[165]
*p*-CA	>	100	µmol/L	IC_50_	Cytotoxicity against human SK-MEL-28 cells after 72 h by MTT assay	SK-MEL-28	[166]
	>	100	µmol/L	IC_50_	Cytotoxicity against human A549 cells after 72 h by MTT assay	A549	[166]
	>	10	µmol/L	IC_50_	Cytotoxicity against human SKOV3 cells after 48 h by SRB assay	SK-OV-3	[164]
	=	10	µmol/L	IC_50_	Cytotoxicity against human BT549 cells after 48 h by SRB assay	BT-549	[164]
	>	2000	µmol/L	IC_50_	Antiproliferative activity against human U937 cells assessed as incorporation of methyl-3H-thymidine after 12 h by scintillation counting	U-937	[167]
	>	200	µmol/L	IC_50_	Antitumor activity against KB cells by MTT assay	KB	[168]
	=	82	µmol/L	IC_50_	Cytotoxicity against human LoVo cells after 72 h by MTT assay	LoVo	[166]
	>	10	µmol/L	IC_50_	Cytotoxicity against human A549 cells after 48 h by SRB assay	A549	[164]
	>	100	µmol/L	IC_50_	Cytotoxicity against human PC3 cells after 72 h by MTT assay	PC-3	[166]
	>	10	µmol/L	IC_50_	Cytotoxicity against human SK-MEL-2 cells after 48 h by SRB assay	SK-MEL-2	[164]
CFA	=	317	µmol/L	IC_50_	Antiproliferative activity against human U937 cells assessed as incorporation of methyl-3H-thymidine after 12 h by scintillation counting	U-937	[167]
	=	700	µmol/L	IC_50_	Cytotoxicity against human A549 cells assessed as reduction in cell viability measured after 48 h by luminescence-based ATP assay	A549	[167]
	=	6.4	µmol/L	IC_50_	Antiproliferative activity against human MOLM13 cells by CellTiter-Glo assay	MOLM-13	[169]
	=	500	µmol/L	IC_50_	Cytotoxicity against human A549 cells assessed as reduction in cell viability measured after 48 h by FMCA assay	A549	[167]
	=	30	µmol/L	IC_50_	Cytotoxicity against human HCT116 cells after 96 h by MTT assay	HCT-116	[170]
	>	10	µmol/L	IC_50_	Antiproliferative activity against human MV4-11 cells by CellTiter-Glo assay	MV4-11	[169]
	=	76	µmol/L	IC_50_	Neuroprotection against amyloid beta (25 to 35)-induced cell death in rat PC12 cells pre-incubated for 3 h followed by amyloid beta addition measured after 24 h by MTT assay	PC-12	[171]
	-	-	-	IC_50_	Activity against hydrogen peroxide induced DNA damage in Jurkat T cells	Jurkat	[172]
	=	27	µmol/L	IC_50_	Cytotoxicity against human HT-29 cells after 96 h by MTT assay	HT-29	[170]
	=	700	µmol/L	IC_50_	Cytotoxicity against human A549 cells assessed as reduction in cell viability measured after 48 h by MTT assay	A549	[167]
	>	550	µmol/L	IC_50_	Cytotoxicity against African green monkey Vero cells assessed as [3H]-hypoxanthine incorporation after 48 h	Vero	[173]
	>	550	µmol/L	IC_50_	Cytotoxicity against human MCF7 cells assessed as [3H]-hypoxanthine incorporation after 48 h	MCF7	[173]
	=	129	µmol/L	IC_50_	Cytotoxicity against human AGS cells after 96 h by MTT assay	AGS	[170]
	=	940	µmol/L	IC_50_	Anticomplement activity in rabbit erythrocytes assessed as concentration required for 50% hemolytic inhibition by alternative pathway pre-treated for 10 min with normal human serum followed by erythrocyte addition measured after 30 min by spectrophotometric method	Erythrocyte	[174]
	=	44.0	µg/mL	IC_50_	Antiallergic activity in Ca(2+)-stimulated differentiated human HeLa cells assessed as inhibition of cys-leukotriene release after 6 days by ELISA	HeLa	[175]
	=	0.002	µmol/L	IC_50_	Antiproliferative activity against human T47D cells after 5 days by MTT assay	T47D	[176]
	=	750	µmol/L	IC_50_	Anticomplement activity in sheep erythrocytes assessed as concentration required for 50% hemolytic inhibition by classic pathway pre-treated for 10 min with guinea pig serum followed by erythrocyte addition measured after 30 min by spectrophotometric method	Erythrocyte	[174]
	>	100	µmol/L	IC_50_	Cytotoxicity against human LNCAP cells assessed as reduction in cell viability after 24 h by WST-1 assay	LNCaP	[177]
	>	100	µmol/L	IC_50_	Antiproliferative activity against human A549 cells after 72 h by MTT assay	A549	[178]
	>	10	µmol/L	IC_50_	Antiproliferative activity against human MOLM14 cells by CellTiter-Glo assay	MOLM-14	[169]
RA	=	40.4	%	Inhibition	Inhibition of Jurkat cell activation assessed as blocking of T-cell antigen receptor-induced IL-2 expression at 10 µmol/L by luciferase assay	Jurkat	[179]
	=	50	%	Inhibition	Inhibition of Jurkat cell activation assessed as blocking of T-cell antigen receptor-induced IL-2 expression at 30 µmol/L by luciferase assay	Jurkat	[179]
	-	-	-	Activity	Cytotoxicity against human HepG2 cells up to 20 µmol/L after 24 h by MTS assay	HepG2	[180]
	=	71	%	Activity	Inhibition of cell proliferation of human U251 cells assessed as cell viability at 100 µmol/L after 72 h by SRB assay	U-251	[181]
	=	27	%	Inhibition	Inhibition of Wnt/beta-catenin signaling pathway in human HEK293 cells at 20 µmol/L after 24 h by dual luciferase reporter gene assay relative to vehicle-treated control	HEK293	[182]
	-	-	-	Activity	Cytoprotection against phototoxicity in human NHDF cells assessed as increase in cell viability at 3.9 to 31.3 µmol/L preincubated for 60 min followed by 7.5 J/cm^2^ UVA irradiation and measured after 24 h by neutral red uptake assay	NHDF	[183]
	-	-	-	Activity	Cytoprotection against phototoxicity in human HaCaT cells assessed as increase in cell viability at 3.9 µmol/L preincubated for 60 min followed by 10 J/cm^2^ UVA irradiation and measured after 24 h by neutral red uptake assay	HaCaT	[183]
	-	-	-	Activity	Cytoprotection against phototoxicity in human NHDF cells assessed as increase in cell viability at 3.9 to 31.3 µmol/L preincubated for 60 min followed by 150 mJ/cm^2^ UVB irradiation and measured after 24 h by neutral red uptake assay	NHDF	[183]
	-	-	-	Activity	Cytoprotection against phototoxicity in human HaCaT cells assessed as increase in cell viability at 3.9 to 31.3 µmol/L preincubated for 60 min followed by 10 J/cm^2^ UVA irradiation and measured after 24 h by neutral red uptake assay	HaCaT	[183]
	=	2.9	µmol/L	IC_50_	Antiproliferative activity against human cells by CellTiter-Glo assay	MOLM-13	[169]
	>	10	µmol/L	IC_50_		MV4-11	[169]
	=	7.1	µmol/L	IC_50_		MOLM-14	[169]
	=	55	µmol/L	CC_50_	Cytotoxicity against human MT4 cells by MTT method	MT4	[184]
D-ChA	=	39.7	µmol/L	IC_50_	Concentration of compound required to reduce MT-4 cell viability by 50%	MT4	[185]
	=	35.5	µmol/L	IC_50_	Compound was evaluated for the cytoprotection of CEM-SS cells by XTT cytoprotection assay through the NCI AIDS Screen	CEM-SS	[185]
L-ChA	=	20.1	µmol/L	IC_50_		CEM-SS	[185]
	=	45	µmol/L	IC_50_	Concentration of compound required to reduce MT-4 cell viability by 50%	MT4	[185]
D-ChA	=	111	µmol/L	CC_50_	Cytotoxicity against human MT4 cells by MTT assay	MT4	[186]

SRB—sulforhodamine B; MTT—3-(4,5-dimethylthiazol-2-yl)-2,5-diphenyltetrazolium bromide; XTT—(2,3-bis-(2-methoxy-4-nitro-5-sulfophenyl)-2H-tetrazolium-5-carboxanilide).
